# Multilocular subcutaneous bacillary angiomatosis as a primary manifestation of AIDS

**DOI:** 10.1002/ski2.454

**Published:** 2024-08-28

**Authors:** Theresa M. Duckwitz, Amir S. Yazdi, David Kluwig

**Affiliations:** ^1^ Clinic for Dermatology and Allergology University Hospital Aachen Aachen Germany; ^2^ Clinic for Geriatrics und Palliative Care Hermann‐Josef‐Krankenhaus Erkelenz Erkelenz Germany

## Abstract

A 34‐year‐old male patient presented with a clinical picture of multilocular subcutaneous skin nodules in addition to marked lymphadenopathy and general physical deterioration. A comprehensive diagnostic workup including serology, skin biopsy and imaging studies led to the initial diagnosis of human immunodeficiency virus (HIV) infection in AIDS stage with rare multilocular subcutaneous bacillary angiomatosis (BA) caused by Bartonella henselae. BA describes a process of neovascularisation of the skin or of internal organs (particularly the liver and spleen) and was first described in HIV‐positive patients by Stoler et al. in 1983. Both cutaneous and systemic symptoms are variable. There is no standardized treatment. The patient was started on antibiotic therapy with doxycycline, which was subsequently augmented with rifampicin. As the patient's general condition deteriorated and lymphocytopenia aggravated, he was transferred to an internal medicine ward for further treatment and subsequently commenced on antiretroviral therapy. This case corroborates numerous aspects of the cases described in the literature yet differs from them in that subcutaneous lesions are uncommon, particularly when infected with Bartonella henselae, illustrating the clinical spectrum of BA. Furthermore, it emphasises the significance of prompt and thorough diagnosis encompassing HIV serology in instances of skin lesions, accompanied by systemic signs and evidence of immunosuppression.

## Graphical abstract

Using the clinical example of bacillary angiomatosis (BA) in the initial diagnosis of HIV infection, this case report illustrates the importance of prompt and comprehensive diagnostic testing for unusual skin changes, particularly when accompanied by general symptoms and indications of immunosuppression. Furthermore, it highlights the diverse clinical manifestations and treatment challenges associated with BA.

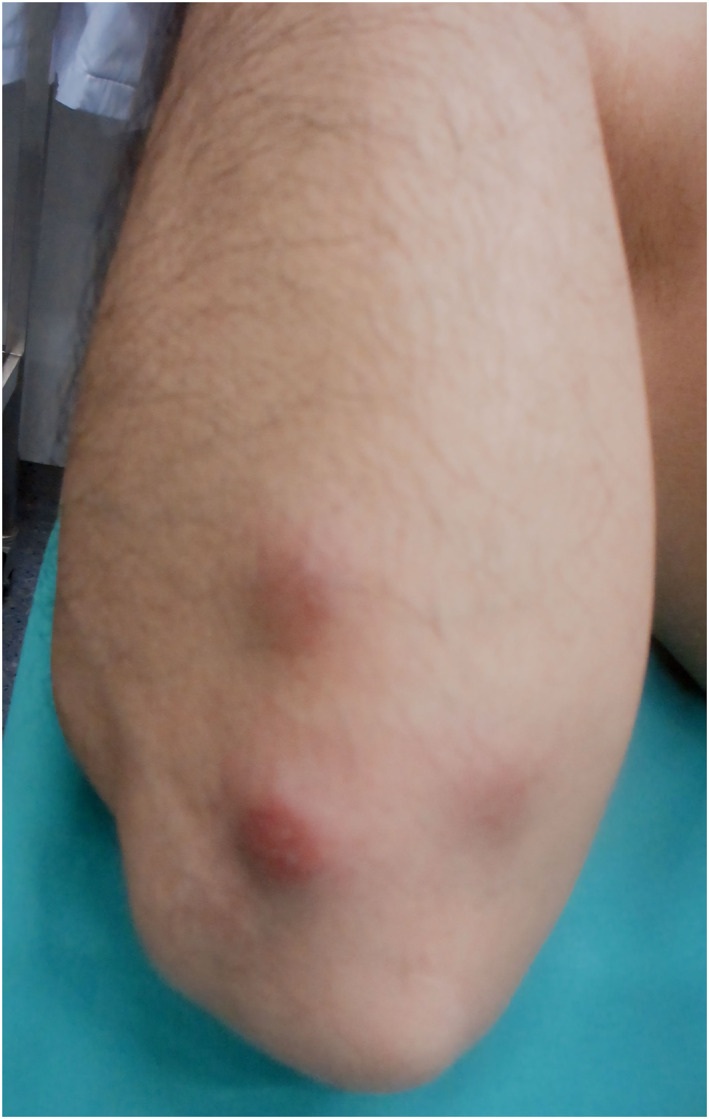

## 1 INTRODUCTION

Bacillary angiomatosis (BA) is a process of neovascularisation of the skin or internal organs (particularly the liver and spleen), caused by aerobic, gram‐negative pathogens, including Bartonella henselae (which causes cat‐scratch disease) and Bartonella quintana.^1^, ^2^ The disease was first described in HIV‐positive patients by Stoler et al. in 1983.^3^ However, it can also occur in other forms of immunosuppression, such as chemotherapy or organ transplantation regimes as well as in immunocompetent patients.^1^, ^2^ At the time of its discovery, BA was described as an atypical subcutaneous infection,^2^ although over the years it has shown a high variability of clinical manifestations. In addition to various cutaneous lesions, regional lymphadenopathy and systemic manifestations, involving the liver, spleen, skeleton, bone marrow, soft tissues and the central nervous system, are possible.^4^

Furthermore, there is clinical heterogeneity in cutaneous manifestations: In BA, skin lesions typically begin as papules that subsequently develop into erythematous or violaceous nodules. These may vary in size and tend to ulcerate and bleed. In the majority of cases, the lower extremities are affected. Mucosal involvement is also possible, including the gastrointestinal tract.^2^

While cat scratch disease, caused by Bartonella henselae, is, as the name suggests, typically transmitted by contact with cats, only 20% of BA cases are associated with preceding cat exposure. It is therefore important to consider other potential sources of infection.^5^

The diagnosis of BA is based on histology and serology. The recommended treatment is doxycycline or macrolides. In cases of severe infection, these may be combined with rifampicin or gentamicin.^2^, ^6^

In our patient, the described skin changes as the main symptom led not only to the diagnosis of BA but also to the initial diagnosis of human immunodeficiency virus (HIV) infection.

## 2 PATIENT AND METHODS

A 34‐year‐old male patient presented to our dermatology clinic with painful and pruritic subcutaneous nodules lasting approximately 2.5 months. Previous treatment with systemic steroids had been unsuccessful. The patient also reported occasional night sweats. The patient came to Germany 14 years ago as a refugee from Afghanistan.

**FIGURE 1:**
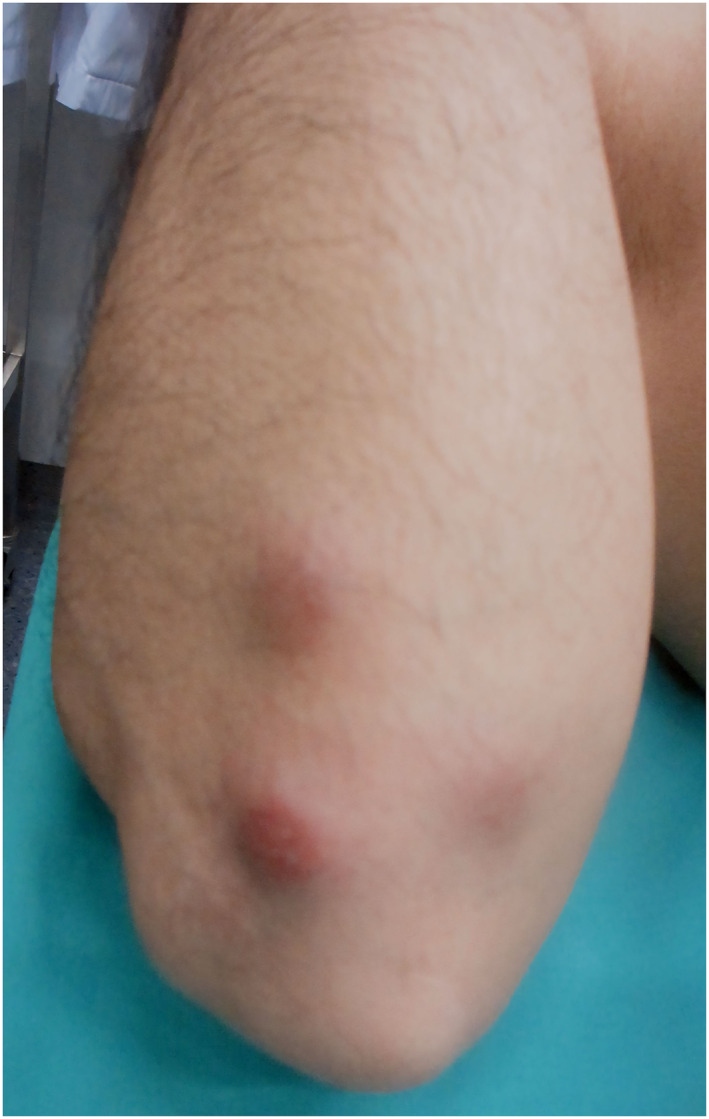
Right forearm with erythematous nodules.

**FIGURE 2:**
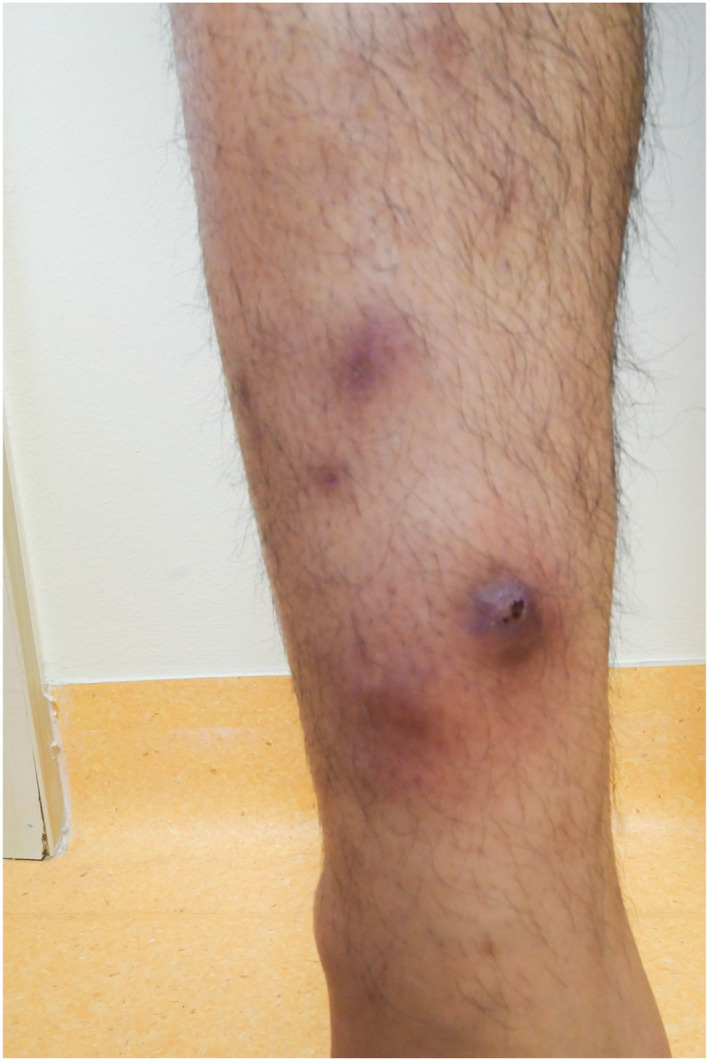
Left lower leg with partly central ulcerated nodi.

On clinical examination, the patient presented with a reduction in his general condition as well as multiple tender subcutaneous nodules on the integument. The nodules were brownish to violaceous and palpable. They were distributed over the extremities (Figures 1 and 2), except for one nodule, which was identified on the abdomen. Additionally, an enlarged palpable lymph node was observed in the right submandibular region.

A chest X‐ray and a lymph node sonography were conducted. The examination revealed the presence of enlarged, morphologically normal cervical and axillary lymph nodes. One of the subcutaneous nodes was excised for histological and microbiological examination. Furthermore, we completed the microbiological diagnostics with serological pathogen tests for relevant viral and bacterial pathogens.

## 3 RESULTS

**FIGURE 3:**
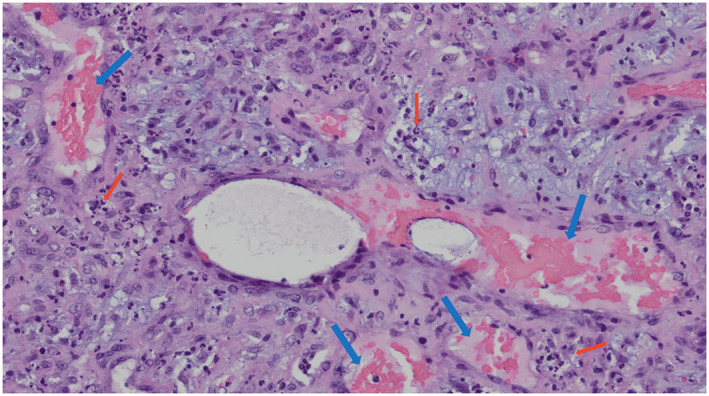
Histological examinations of the subcutaneous nodule revealed a vascular tumour (blue arrows) with masses of neutrophils (red arrows).

The patient was diagnosed with HIV, as evidenced by a positive HIV screening test and an elevated HIV‐1 viral load. At the time of diagnosis, CD4‐positive T‐cells were severely reduced (45 cells/μl). Histological examinations (Figure 3) of the subcutaneous nodule revealed a vascular tumour (blue arrows) with masses of neutrophils (red arrows). Furthermore, Bartonella DNA was identified in the tissue by PCR. Serological testing demonstrated elevated Bartonella henselae IgG, confirming the diagnosis of BA.

For the treatment of BA, we administered doxycycline 100 mg orally twice a day.

As the disease progressed, the patient's general condition further deteriorated with recurrent fever, hypotension and tachycardia. An emergency computed tomography (CT) scan revealed Pneumocystis jirovecii (PJ) pneumonia. In addition, rifampicin 300 mg was administered intravenously twice daily, and in the presence of clinical signs of sepsis and marked lymphocytopenia (0.14/nL), ceftriaxone 2 g was added intravenously once a day. For further treatment, the patient was transferred to the clinic for pneumology and internal intensive care where a high‐dose administration of cotrimoxazole was initiated for a confirmed case of PJ pneumonia.

Over time, the patient's general condition improved and antiretroviral therapy with Truvada (emtricitabine and tenofovir) and Tivicay (dolutegravir) was started. Ultimately, the patient was discharged for outpatient follow‐up.

## 4 DISCUSSION

The presented case illustrates the necessity of a comprehensive diagnostic workup for unclear, progressive skin conditions. Targeted infectious diagnostics in the setting of initial cutaneous manifestations could identify a severe underlying disease with relevant immunosuppression.

A distinctive feature of our case is the cutaneous presentation of BA in the form of subcutaneous nodules. A PubMed search revealed that, while there are numerous documented cases of BA presenting with a variety of cutaneous lesions, only four with subcutaneous nodules could be identified.^7^, ^8^, ^9^, ^10^ Mohle‐Boetani et al. observed that 24% of 42 patients with BA had subcutaneous alterations.^11^

The diagnostic summary (elevated Bartonella henselae IgG titre and positive Bartonella DNA in the PCR from biopsy tissue), indicates a probable infection with Bartonella henselae, although further characterisation of the detected Bartonella henselae DNA would have been beneficial. It is noteworthy that a strong association between Bartonella quintana and subcutaneous lesions has been documented in the scientific literature. Conversely, B. henselae infection is more frequently associated with peliosis of the liver and spleen in addition to cutaneous lesions.^2^, ^12^ In our case, however, while the patient's CT scan was unremarkable with respect to the liver and spleen, the cutaneous lesions were also consistent with a Bartonella quintana infection, although it was not detected serologically.

The case presented by us shares similarities with patients described in the literature. These include a very low CD4‐positive T‐cell count (e.g.^1^, ^13^), lymphadenopathy^2^, ^4^, ^14^ and vascular histology.^6^, ^14^

Given that there is currently no standardized treatment regimen for BA, the medication given to our patient can only be compared with recommendations from individual reports. The predominant treatment is doxycycline or macrolides, in combination with rifampicin or gentamicin depending on the severity of the infection.^6^

The relatively late initial diagnosis of HIV in AIDS stage may appear to be an exceptional case, given that, with a severely reduced CD4 cell count (below 200 per μl) and with PJ pneumonia as an AIDS‐defining disease at initial diagnosis, our patient falls into the group of ‘late presenters’. However, research conducted by Valbert et al. indicates that this group accounts between 44% and 64% of all new HIV diagnoses in Germany and across Europe.^15^ These figures highlight the necessity of striving for an earlier initial diagnosis of HIV.

## 5 CONCLUSION

The presented case illustrates the importance of prompt and comprehensive diagnostic testing for unusual skin changes, particularly when accompanied by systemic symptoms and indications of immunosuppression. Given the persistent proportion of ‘late presenters’, HIV infection must be considered in all cases.

This case highlights the diverse clinical manifestations and treatment challenges associated with BA, particularly in the context of an underlying immunosuppressive condition. The early administration of anti‐infective therapy is of paramount importance for the patient's prognosis.

## CONFLICT OF INTEREST STATEMENT

The authors declare that the research was conducted in the absence of any commercial or financial relationships that could be construed as a potential conflict of interest.

## AUTHOR CONTRIBUTIONS

**Theresa M. Duckwitz**: Conceptualisation (lead); data curation (lead); formal analysis (lead); funding acquisition (equal); investigation (lead); methodology (lead); project administration (lead); resources (equal); software (equal); supervision (supporting); validation (equal); visualisation (equal); writing—original draft (lead); writing—review and editing (equal). **Amir S. Yazdi**: Conceptualisation (supporting); data curation (supporting); formal analysis (supporting); funding acquisition (equal); investigation (supporting); methodology (equal); project administration (supporting); resources (equal); software (equal); supervision (equal); validation (equal); visualisation (equal); writing—original draft (supporting); writing—review and editing (equal). **David Kluwig**: Conceptualisation (supporting); data curation (supporting); formal analysis (supporting); funding acquisition (equal); investigation (supporting); methodology (equal); project administration (supporting); resources (equal); software (equal); supervision (equal); validation (equal); visualisation (equal); writing—original draft (supporting); writing—review and editing (equal).

## ETHICS STATEMENT

No experiments on humans or animals were undertaken for this study. Based on the purely retrospective description and literature research, no ethics vote is available. The current version of the Declaration of Helsinki was respected.

## PATIENT CONSENT

Informed consent was obtained from the reported patient after verbal and written informed consent.

## Data Availability

The data that support the findings of this study are available from the corresponding author upon reasonable request.
